# Effectiveness of Postoperative Adjuvant Radiotherapy in Atypical Meningioma Patients After Gross Total Resection: A Meta-Analysis Study

**DOI:** 10.3389/fonc.2020.556575

**Published:** 2021-01-15

**Authors:** Lingzhe He, Buyi Zhang, Jianmin Zhang, Zhige Guo, Feina Shi, Qiang Zeng

**Affiliations:** ^1^Department of Neurosurgery, Second Affiliated Hospital of Zhejiang University School of Medicine, Hangzhou, China; ^2^Department of Neurology, Sir Run Run Shaw Hospital of Zhejiang University School of Medicine, Hangzhou, China

**Keywords:** atypical meningioma, radiotherapy, gross-total resection, surgery, prognosis

## Abstract

**Background:**

It still remains unclear whether patients with atypical meningioma (AM) could benefit from postoperative adjuvant radiotherapy (PORT) after gross-total resection (GTR).

**Objective:**

Exploring the effectiveness of PORT on AM patients after GTR.

**Methods:**

Literatures on PubMed, Embase, Web of science, and Scopus databases published between January 2000 and January 2019 were searched. After the selection based on the certain exclusion criteria, the Newcastle-Ottawa evaluation scale was used to evaluate the quality of the included literatures. Finally, a meta-analysis was conducted to analyze the effectiveness of PORT on local control (LC), progression-free survival (PFS) and overall survival (OS) in atypical meningioma patients after GTR.

**Results:**

A total of 17 articles with 2,008 AM patients were included in the meta-analysis. The 5-year LC, 5-year PFS, and 5-year OS rates were 82.2, 84.1, and 79.0%, respectively, for AM patients receiving PORT after GTR, and they were 71.0, 71.9, and 81.5%, respectively, for those not receiving PORT after GTR. PORT could significantly improve 5-year LC rate (OR [95% Cl] = 2.59 [1.40–4.81], *P* = 0.002) and 5-year PFS rate (OR [95% Cl] = 1.99 [1.35–2.95], *P* = 0.001), but did not significantly improve 5-year OS rate (OR [95% Cl] = 1.07 [0.60–1.91], *P* = 0.828).

**Conclusion:**

PORT could improve the 5-year LC rate and 5-year PFS rate in AM patients after GTR. AM patients might benefit from PORT after GTR.

## Introduction

Meningiomas are the most common primary intracranial tumors with an incidence rate of about 8 per 100,000 population, accounting for approximately 37% of all central nervous system tumors (1). According to WHO 2016 classification, it can be divided into WHO grades I–III (2).

Compared to benign meningiomas (WHO grade I), atypical meningiomas (WHO grade II) have a more aggressive behavior, a higher risk of recurrence (seven to eight times increased in 5 years) and a higher mortality (3–5). Therefore, it is particularly important to find out the factors which could significantly influence the prognosis of AM patients. The common consensus is that postoperative adjuvant radiotherapy (PORT) is generally recommended for meningioma patients underwent subtotal resection (STR). Whether AM patients need PORT after GTR depends on the grade of meningiomas (6). After GTR, follow-up observation is generally recommended for benign meningioma patients, while adjuvant radiotherapy is routinely recommended for malignant meningioma patients (WHO grade III) (6). But there is still a controversy for atypical meningioma (AM) patients because of the unclear effectiveness of PORT. Several studies with small sample sizes have been performed to investigate the effect of PORT in AM patients after GTR, but obtained contradictory results (7–25). A recent study based on the National Cancer Database found that PORT and GTR were both associated with improved survival for AM patients (26). Whereas our recent study based on the Surveillance, Epidemiology, and End Results database found that PORT might not prolong the overall survival (OS) in AM patients undergoing GTR (27).

Hasan et al. published a meta-analysis concerning the efficacy of PORT after GTR on AM patients in 2014 (28). That study showed that for the enrolled 757 patients, PORT significantly reduced the risk of recurrence and increased the local control rate for 5 years, but did not reduce the overall mortality (28). However, the articles included in this meta-analysis were published between 1993 and 2013. Besides, they did not analyze the impact of PORT on progression-free survival (PFS), which is also an important prognostic indicator. With the great modifications in the 2000 WHO classification criteria for meningioma and a large number of articles focused on the prognosis of AM patients after GTR in recent years, it is necessary to summarize them again. The aim of this study was to systematically review and meta-analyze the effectiveness of PORT in AM patients after GTR.

## Materials and Methods

### Literature Search and Study Selection

A systematic review of the literatures on the relationship between PORT and the prognosis of AM patients after GTR between January 2000 and January 2019 in the Pubmed, Embase, Web of science, and Scopus databases was performed. The search of published articles was undertaken using the following terms: “gross total resection,” “atypical meningioma” or “grade II meningioma” and “radiotherapy.”

The excluding criteria are listed as below: (1) research subjects were not well defined, such as AM patients including benign meningioma patients or malignant meningioma patients, and GTR patients including STR patients; (2) the efficacy of PORT after GTR on AM patients was not compared; (3) AM was defined according to the WHO classification criteria before the year 2000; (4) the 5-year prognostic data were not available; (5) non-English literatures or literatures of systematic reviews, case reports, observational studies; (6) database-based researches.

### Data Extraction and Quality Assessment

Two investigators extracted data from the relevant articles independently. If the opinions or data were inconsistent, they would discuss until consensus were reached. Extracted data should include: name, year of publication, the type of study, WHO classification criteria, the number of cases, age, gender, the grade of meningioma, degree of surgical resection, PORT, and the treatment endpoint. GTR was defined as Simpson grade I-III in this study.

Newcastle-Ottawa Scale (NOS) literature quality assessment scale and revised standards were used to assess quality of included articles. The evaluation scales were based on the following three indicators: patient selection, study comparability, and research outcome. The score was 9 points in total, the article with 6 points or more was considered as high-quality.

### Statistical Analysis

The patients were divided into two groups, patients received GTR plus PORT and patients received GTR without PORT. Because of the cumulative survival rates, we performed a meta-analysis by converting that to the assumed cumulative number of survivors. Odds ratios (ORs) with 95% confidence interval (CI) were utilized to evaluate the difference in 5-year local control (LC) rate, 5-year PFS rate, and 5-year OS rate between the two groups. Heterogeneity of pooled results was assessed using Cochrane’s Q test and *I^2^* measurement. *P* > 0.10 or *I^2^* < 50% indicated that the heterogeneity was not significant, and then a fixed-effects model was used. Otherwise, a random effect model was used. Sensitivity analysis was conducted to evaluate the validity and reliability of present meta-analysis. Begg’s funnel plot and Egger’s test were used to evaluate the publication bias risk. All statistical analyses were performed using STATA version 15.1 (STATA Corporation, College Station, TX, USA), and all *P* values were two sides.

## Results

### Study Characteristics

**Figure 1:**
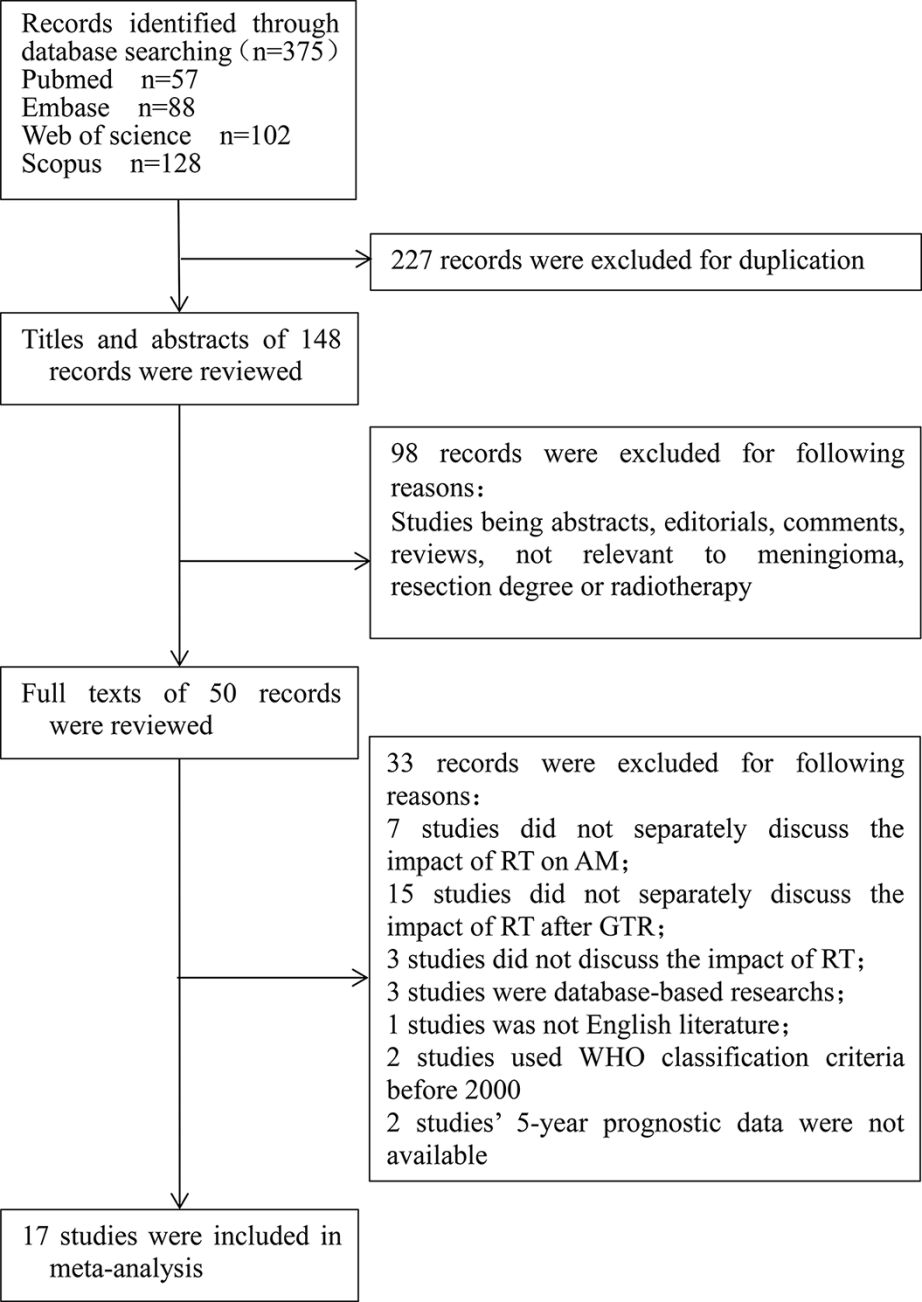
Flow chart of study selection procedure.

According to the above search strategy, 273 articles were initially screened. The detailed screening process was shown in **Figure 1**. By carefully reading the literature titles and abstracts, and excluding the literature of which types or contents did not meet research topics, 50 articles were initially included. By reading the full text, 33 articles were excluded according to the excluding criteria. Finally, a total of 17 articles were included in this meta-analysis.

The basic characteristics of the final included studies were shown in **Table 1**. The data included the author, the year of publication, the country, the year of treatment, the WHO classification standard, the type of study, the sample data, and the survival rate. A total of 2,008 AM patients were included in the 17 articles. Among them, 1,492 patients did not receive PORT after GTR and 369 patients received PORT after GTR.

**Table 1:** Characteristics of studies for association between PORT and survival.

Author/year of publication/country	Year of treatment	WHO classification criteria	Type of study	No. of case	Sex(male/female)	Age(year)	Follow-up time(month)	GTRdefinition	No. of GTR	No. of PORT	Indicator	NOS	
Manish K. Aghi/2009/USA (8)	1993–2004	2000	Retrospective cohort study	108	48/69	55 (19–82)	39 (1–168)	Simpson grade I resection	80	8	OR、5-year LC	8/9	
Kyungil Jo/2010/Korea (9)	1997–2008	2000	Retrospective cohort study	35	18/17	56 (15–80)	40 (6–97)	Simpson grade I resection	32	19	OR、5-year LC	7/9	
Kangmin D. Lee/2013/USA (10)	1999–2009	2007	Retrospective cohort study	90	34/56	56.9 (22–83)	48.7 (12–108)	Simpson grade I–III resection	71	17	OR、5-year PFS	8/9	
Sam Q. Sun/2014/USA (11)	1993–2012	2007	Retrospective cohort study	151	63/88	54 (8.8–86.2)	45 (6–232)	Simpson grade I–III resection	151	39	OR、5 year LC+PFS	8/9	
Michael D. Jenkinson/2016/UK (12)	2001–2010	2000/2007	Retrospective cohort study	133	68/65	62 (22–86)	57.4 (0.1–152.2)	Simpson grade I–III resection	113	32	OR、5-year PFS+OS	8/9	
Christopher S. Graffeo/2017/USA (13)	1988–2011	2016	Retrospective cohort study	69	25/44	61 (27–91)	74	Simpson grade I–III resection + no residue in image	69	8	OR、5-year PFS+OS	7/9	
Shakir I. Shakir/2018/Canada (14)	1992–2013	2007	Retrospective cohort study	70	32/38	62 (32–87)	69 (5.2–273.5)	Simpson grade I–III resection	40	12	OR、5-year PFS	8/9
Salah Hammouche/2014/UK (15)	1996–2009	2007	Retrospective cohort study	79	43/36	58 ± 13.6	50 (1–172)	Simpson grade I resection	34	9	OR、5-year PFS	7/9
Hae Jin Park/2013/USA (16)	1997–2011	2000/2007	Retrospective cohort study	83	33/50	52 (24–78)	43 (6.2–160.0)	Simpson grade I–II resection	55	17	5-year PFS	7/9
Richard Mair/2011/UK (17)	2001–2010	2000	Retrospective cohort study	114	55/59	57 (17–85)	NR	Simpson grade I–II resection	66	15	5-year PFS	7/9
Ming Zhi/2018/USA (18)	2002–2012	2000/2007	Retrospective cohort study	149	74/75	64 (18–91)	74.2(0.5–182.2)	Simpson grade I–III resection	98	26	OR、5-year PFS+OS	8/9
Ammoren Dohma/2017/USA (19)	1993–2014	2007	Retrospective cohort study	115	45/70	63.6 ± 14.7	NR	Simpson grade I–III resection	78	41	5-year OS	6/9
Karol P. Budohoski/2018/UK (20)	2007–2014	2016	Retrospective cohort study	220	98/122	61(50–68)	NR	Simpson grade I–III resection	143	35	OR	6/9
Hannah Yoon/2015/USA (21)	2000–2010	2000	Retrospective cohort study	158	72/86	58 (19–90)	NR	Simpson grade I–III resection	109	7	5-year OS	7/9
Yu-Chi Wang/2014/China (22^)^	2001–2009	2007	Retrospective cohort study	28	13/15	56.8 (23–85)	57.4 (16–144)	Simpson grade I–III resection + no residue in image	14	3	5-year PFS	8/9
Charles Champeaux/2016/UK (23)	2000–2015	2000/2007	Retrospective cohort study	178	81/97	57 (44.7–68.8)	43.2 (18–74.4)	Simpson grade I–III resection	127	24	5-year LC	8/9
Douglas A. Hardesty/2013/USA (24)	1992–2011	2007	Retrospective cohort study	228	97/131	62(2–94)	52	Simpson grade I–III resection	149	72	5-year PFS	7/9

OR, overall number of recurrence.

According to the NOS literature quality evaluation scale and revised standards, the included literatures were evaluated. As shown in **Table 1**, the quality of the literatures was generally high (6–9 points).

### Effectiveness of Postoperative Adjuvant Radiotherapy on 5-Year Progression-Free Survival

**Figure 2:**
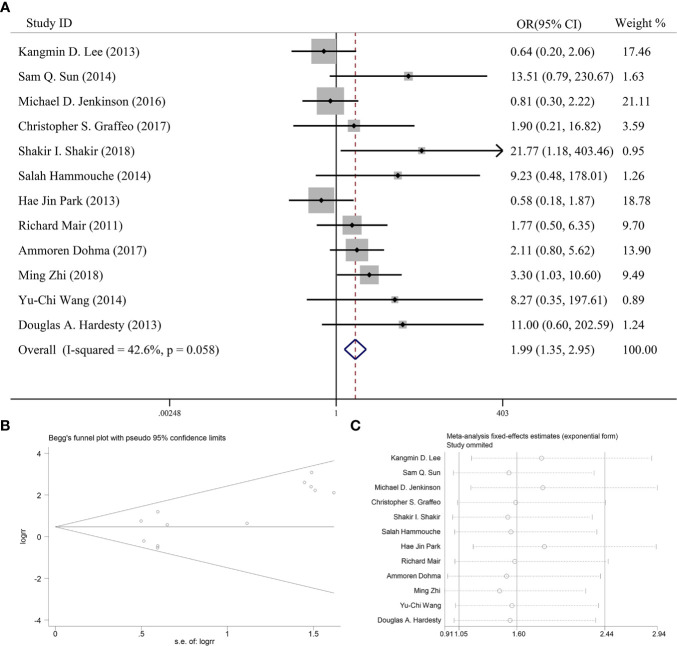
**(A)** Forest plot for the relationships between PORT and 5-year PFS. **(B)** Begg’s funnel plots of publication bias for meta-analysis of PORT. **(C)** Sensitivity analysis for meta-analysis of PORT.

Twelve articles reported the effect of PORT on 5-year PFS in AM patients after GTR. One of the articles suggested that PORT significantly improved 5-year PFS (14), but 11 articles found that PORT had no significant relationship with 5-year PFS. Integrating the above literature data for analysis, 662 AM patients did not receive PORT after GTR, and 276 patients received PORT after GTR. There was no significant difference in heterogeneity analysis (*I^2^* = 42.6%, *P* = 0.058). The 5-year PFS was 84.1% in the patients receiving PORT and 71.9% for those not. The meta-analysis showed that PORT could significantly improve 5-year PFS in AM patients after GTR (OR [95% Cl] = 1.99 [1.35–2.95], *P* = 0.001) (**Figure 2A**). **Figure 2B** indicated that there was no significant publication bias (*P* = 0.075). The sensitive analysis was performed by removing studies one by one, and the removal of any individual study did not affect its overall trend, indicating that the results of this meta-analysis were stable and reliable (**Figure 2C**).

### Effectiveness of Postoperative Adjuvant Radiotherapy on 5-Year Overall Survival

**Figure 3:**
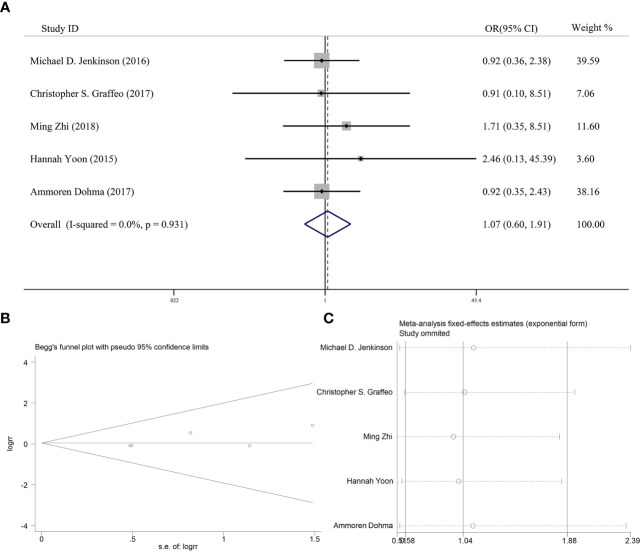
**(A)** Forest plot for the relationships between PORT and 5-year OS. **(B)** Begg’s funnel plots of publication bias for meta-analysis of PORT. **(C)** Sensitivity analysis for meta-analysis of PORT.

Five articles reported the effect of PORT on 5-year OS in AM patients after GTR. None of them found that PORT could significantly improve 5-year OS. Among them, 353 AM patients did not receive PORT after GTR, and 114 patients received PORT after GTR. There was no significant difference in heterogeneity analysis (*I^2^* = 0%, *P* = 0.931). The 5-year OS was 79.0% in the patients receiving PORT and 81.5% in those not. The meta-analysis showed that PORT had no significant relationship with 5-year OS (OR [95% Cl] = 1.07 [0.60–1.91], *P* = 0.828) (**Figure 3A**). **Figure 3B** indicated that there was no significant publication bias (*P* = 0.142). The sensitive analysis was performed by removing studies one by one, and the removal of any individual study did not affect its overall trend, indicating that the results of this meta-analysis were stable and reliable (**Figure 3C**).

### Effectiveness of Postoperative Adjuvant Radiotherapy on 5-Year Local Control

**Figure 4:**
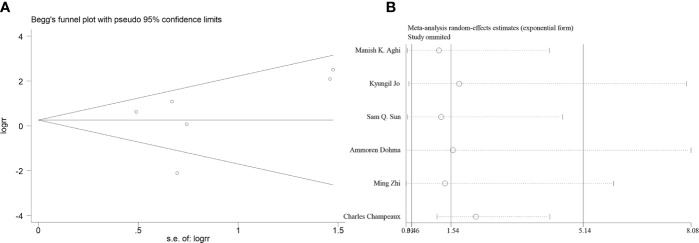
**(A)** Begg’s funnel plots of publication bias for meta-analysis of PORT on 5-year LC before excluding. **(B)** Sensitivity analysis for meta-analysis of PORT.

**Figure 5:**
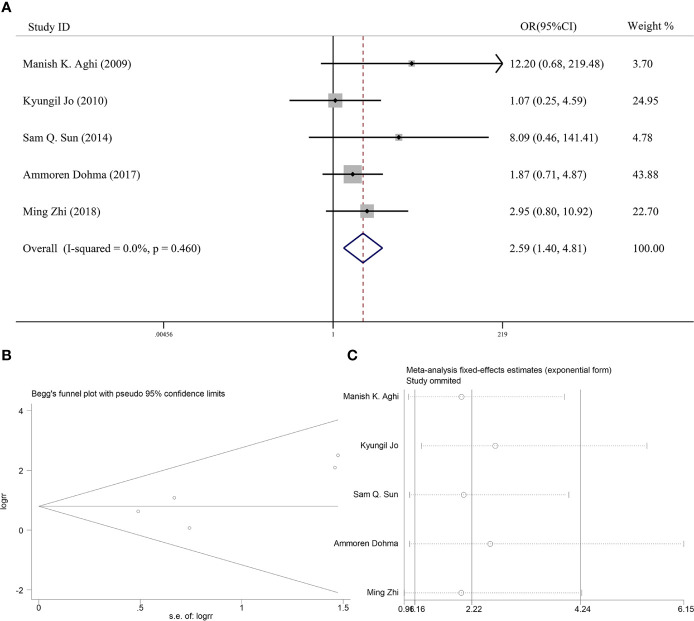
**(A)** Forest plot for the relationships between PORT and 5-year LC after excluding. **(B)** Begg’s funnel plots of publication bias for meta-analysis of PORT. **(C)** Sensitivity analysis for meta-analysis of PORT.

Six articles reported the effect of PORT on 5-year LC in AM patients after GTR. Heterogeneity analysis found significant differences between two groups. Begg’s funnel plot and Egger’s test showed that there was a clear publication bias in an article (**Figures 4A, B**). The sensitive analysis was performed by removing studies one by one, and the Charles Champeaux’s was significantly heterogeneous, so it was excluded. Remaining articles had no heterogeneity (*I^2^* = 0%, *P* = 0.460) (**Figure 5B**). The 5-year LC was 82.2% in the patients receiving PORT and 71.0% for those not. The meta-analysis showed that PORT could significantly improve 5-year LC (OR [95% Cl] = 2.59 [1.40–4.81], *P* = 0.002) (**Figure 5A**). There was no significant publication bias (*P* = 0.142) and the results were stable and reliable (**Figure 5C**).

## Discussion

At present, optimal postoperative management for AM patients after GTR remains a great deal of controversy. The NCCN Guideline recommends radiotherapy in several situations (29), but there is no conclusion on whether radiotherapy is needed for AM patients after GTR. In the current study, we found that PORT could improve the 5-year LC rate and 5-year PFS rate in AM patients after GTR, indicating that AM patients might benefit from PORT after GTR.

PORT plays an important role in post-operative management of AM patients. It was showed that patients without PORT after STR had worse PFS (16). Recently, Chenyang Wang et al. (30) found that PORT could significantly improve OS in AM patients who underwent STR (30), which was consistent with our previous finding (27). Currently, PORT has been routinely recommended for AM patients who underwent STR (6, 31).

However, it is contentiously debated whether AM patients can benefit from PORT after GTR. In recent years, a large number of studies have been conducted to find out whether PORT has an effect on the prognosis of AMs after GTR, but lead to contradictory conclusions. Recently, Shakir et al. performed a single-center retrospective study and reported that PORT could improve the 5-year PFS rate in AM patients after GTR (14). However, many studies revealed the ineffectiveness of PORT on AMs after GTR, although most of them showed that PORT had a trend to improve the 5-year PFS rate (11, 13, 15, 17–19, 22, 24). The non-significant differences may due to the relatively small sample sizes of these studies. Therefore, we performed this meta-analysis with a total of 2,008 AMs enrolled to overcome this limitation. The results showed that PORT could significantly improve 5-year PFS rate and 5-year LC, indicating that AMs can benefit from PORT after GTR.

A previous meta-analysis by Hasan et al., including 14 retrospective studies reported from 1984 to 2012, found that PORT for AM patients might decrease risk for relapse (28). However, the WHO classification standard for meningiomas was greatly modified in 2000. Therefore, the literatures enrolled in the current study were all using 2000 WHO classification criteria or later, and the literatures using WHO classification criteria before 2000 or without identified WHO classification criteria were all excluded. In some degree, our study might reduce sample selection bias. Therefore, we considered it was meaningful to conduct this meta-analysis to show that AM patients could benefit from PORT after GTR, which was in line with that previous meta-analysis.

As reported in the previous literatures, many types of radiotherapy, such as conventional radiotherapy, single-fraction stereotactic radiosurgery, fractionated stereotactic radiotherapy, three-dimensional conformal radiotherapy and intensity modulated radiotherapy, have been conducted in AM patients after GTR (8, 16, 18, 24). However, most of the enrolled articles did not compare the differences in the treating effect among different radiotherapy methods. At present, it still remains unclear which type of radiotherapy is preferred for AM patients after GTR. Further studies are needed to investigate the effect of different types of PORT, which might guide the selection of PORT type for radiotherapist.

Our study also has several limitations. First, all enrolled articles are retrospective single-center studies (8–24), and inherent limitations exist in this kind of studies, such as selection bias. Second, the WHO classification criteria (13) and the definition of GTR (8, 9, 15–17) are somewhat different among the articles which may also lead to bias. Third, in the current meta-analysis, we could not perform multivariable analysis including other factors, such as MIB-1 index, location, brain edema, and several molecular markers, which might also influence tumor behavior and recurrence rate. Forth, we chose 5-year PFS, LC, and OS as the prognostic indicators, because most of previous studies did not reported the rates of PFS, LC, and OS with a longer time. The non-significant effect of PORT on OS in our study might due to the short follow-up time. Thus, a longer window than 5 years might be more effective at teasing out potential advantages of PORT on OS. Further studies with a longer follow-up time are warrant. Thus, further multi-center prospective studies of a large sample size with a panel of these markers and longer follow-up time are needed to confirm our findings. Currently, two phase-III clinical trials, ROAM-EORTC 1308 and NRG-BN003, are now ongoing to investigate whether AM patients can benefit from PORT after GTR.

## Conclusion

This meta-analysis showed that PORT could significantly improve 5-year LC and 5-year PFS in AM patients after GTR, indicating that AM patients may benefit from PORT after GTR. The results from two ongoing phase-III clinical trials (ROAM-EORTC 1308 and NRG-BN003) will further help to address the controversy about the effectiveness of PORT in atypical meningioma patients after GTR.

## Data Availability Statement

The datasets presented in this study can be found in online repositories. The names of the repository/repositories and accession number(s) can be found in the article/supplementary material.

## Author Contributions

All authors listed have made a substantial, direct, and intellectual contribution to the work and approved it for publication.

## Funding

This work was supported by the National Natural Science Foundation of China (81801654) and Zhejiang Provincial Natural Science Foundation (LY20H160037).

## Conflict of Interest

The authors declare that the research was conducted in the absence of any commercial or financial relationships that could be construed as a potential conflict of interest.
